# Metal Oxide Nanostructures (MONs) as Photocatalysts for Ciprofloxacin Degradation

**DOI:** 10.3390/ijms24119564

**Published:** 2023-05-31

**Authors:** Petronela Pascariu, Carmen Gherasim, Anton Airinei

**Affiliations:** Petru Poni Institute of Macromolecular Chemistry, 41A Grigore Ghica Voda Alley, 700487 Iasi, Romania; dorneanu.petronela@icmpp.ro (P.P.); gherasim.carmen@icmpp.ro (C.G.)

**Keywords:** metal oxide nanostructures (ZnO; TiO_2_; CuO), materials preparations, enhanced photocatalytic activity, ciprofloxacin degradation

## Abstract

In recent years, organic pollutants have become a global problem due to their negative impact on human health and the environment. Photocatalysis is one of the most promising methods for the removal of organic pollutants from wastewater, and oxide semiconductor materials have proven to be among the best in this regard. This paper presents the evolution of the development of metal oxide nanostructures (MONs) as photocatalysts for ciprofloxacin degradation. It begins with an overview of the role of these materials in photocatalysis; then, it discusses methods of obtaining them. Then, a detailed review of the most important oxide semiconductors (ZnO, TiO_2_, CuO, etc.) and alternatives for improving their photocatalytic performance is provided. Finally, a study of the degradation of ciprofloxacin in the presence of oxide semiconductor materials and the main factors affecting photocatalytic degradation is carried out. It is well known that antibiotics (in this case, ciprofloxacin) are toxic and non-biodegradable, which can pose a threat to the environment and human health. Antibiotic residues have several negative impacts, including antibiotic resistance and disruption of photosynthetic processes.

## 1. Introduction

Nanotechnology offers groundbreaking solutions for water treatment across various areas such as catalysis, adsorption, electrostatics, reactivity, and the ability to control pore volume. Nanomaterials have been shown to be highly effective in the removal of organic and inorganic pollutants and heavy metals from wastewater. In nanomaterials, physical properties are modified by improving volume-to-surface ratios and the effect of quantum properties on particle size. Likewise, the magnetic, optical, and electrical properties of nanostructures are significantly different compared to conventional materials. Moreover, characteristics such as high adsorption, catalytic activity, and reactivity are associated with nanomaterials. Nanomaterials are promising tools for application in various wastewater ecosystems, which include carbon nanotubes, zerovalent nanoparticles, metal oxide nanoparticles, nanocomposites, etc. [1].

Metal oxide nanostructures have gained much attention due to their particular physical and chemical properties, and applications in different fields of industry (sensors, actuators, field-emission devices, solar cells, ultra-violet laser diodes, photocatalysts, spintronic and piezoelectric devices, medicine, etc.) [2]. The rapidly advancing field of nanotechnology has an impact in several areas interfacing with life and physical sciences. The functional properties of materials can change significantly when their dimensions are reduced from micrometric to nanometric. It is well known that electrical conductivity, mechanical properties, active surface, chemical reactivity, and biological activity can change depending on the size of the materials [2].

The fabrication of metal oxide nanoparticles using high-efficiency and low-cost biological sources is of interest to chemists, biologists, and material scientists. Metal oxide nanostructures including TiO_2_, ZnO, CuO, WO_3_, CeO_2_, AgO, and SnO_2_ possess unique characteristics such as magnetic and hydrophobic properties [3]. These types of nanostructures present potential applications in catalysis to convert hydrocarbons into carbon dioxide, as well as in nanoenergetics. Moreover, these nanostructured materials have been used as photocatalysts or as hybrids coupled/doped with other materials to facilitate the degradation of organic pollutants such as pesticides, dyes, drugs, and polycyclic aromatic hydrocarbons [4,5,6]. It has been of particular interest to the scientific community to use metal oxide photocatalysts for antibiotic degradation since they absorb light efficiently under UV or visible light. In addition, these materials are biocompatible, safe, and stable under different test conditions [7,8,9]. Several studies showed that oxide semiconductor materials have some challenges regarding their ineffectiveness, the low exploitation of visible light, or the low absorption ability of some pollutants due to their wide bandgap and faster electron–hole recombination [10,11]. For example, TiO_2_ is one of the most commonly used metal oxide semiconductors for photocatalysis due to its good optical and electronic properties, chemical stability and reusability, non-toxicity, and inexpensive [12]. Additionally, ZnO is another semiconductor material with better quantum efficiency and higher photocatalytic efficiency as compared to TiO_2_, if used for the photocatalytic degradation of antibiotics at neutral pH. In contrast, without any structural modification, the high recombination rate of the photogenerated electron–hole pairs limits the use of pure ZnO [13]. Numerous research studies have proven that the doping of zinc oxide (ZnO) with metals such as silver (Ag) and iron (Fe), as well as non-metals such as nitrogen (N) and carbon (C), leads to higher effectiveness in the degradation of antibiotics through photocatalytic processes [14,15]. In this context, CuO has garnered significant interest in recent years owing to its affordability and non-toxic nature, making it a highly promising candidate [16].

According to their specific applications, the chemical composition (size, shape, morphology, crystallinity) and structural properties of engineered nanomaterials are of great importance. Therefore, the synthesis method should selectively yield the desired properties of the nanostructures to a great extent. Moreover, the metal oxide nanostructured materials can be obtained by a variety of synthesis methods, which include co-precipitation, hydrothermal, sol–gel process, chemical vapor deposition, sonochemical and biogenic routes, electrochemical method, electrospinning technique, etc. [17,18,19,20].

In the last decades, industrial developments have led to massive contamination of the environment, which affects human and aquatic life. Industrial wastes produced by chemical, textile, metallurgical, and mining industries, and pharmaceutical fields are largely responsible for water contamination. Pharmaceutical products are among the most harmful contaminants of wastewater, including antibiotics which are the most dangerous organic compounds due to their wide range of use in both human and veterinary medicine [21]. Among the most common antibiotics found in wastewater, ciprofloxacin, which is a fluoroquinolone antibiotic effective against Gram-positive and Gram-negative bacteria, has a top position. This has been identified in wastewater treatment effluents in the concentration range of 0.2–1 μg/L [22]. Therefore, conventional wastewater treatment methods cannot completely remove this type of pollutant. Likewise, the presence of this antibiotic in aquifers could contribute to the development of antimicrobial resistance. In this way, the need to develop an appropriate technology for the elimination of antibiotics (in the present case ciprofloxacin) is a priority for the scientific world. In this context, heterogeneous photocatalysis has proven to be a satisfactory strategy for the removal of these organic pollutants.

Recently, photocatalysts based on metal oxide semiconductor nanostructures have gained considerable attention due to their potential contribution to environmental problems. Therefore, the main objective of this review is to summarize the scientific progress made in obtaining these semiconductor materials, the improvement of the photocatalytic performance of the main metal oxide semiconductors, and to study the photocatalytic degradation mechanisms of ciprofloxacin in the presence of these materials.

## 2. Methods of Obtaining Oxide Semiconductor Nanostructures Used as Photocatalysts

**Figure 1:**
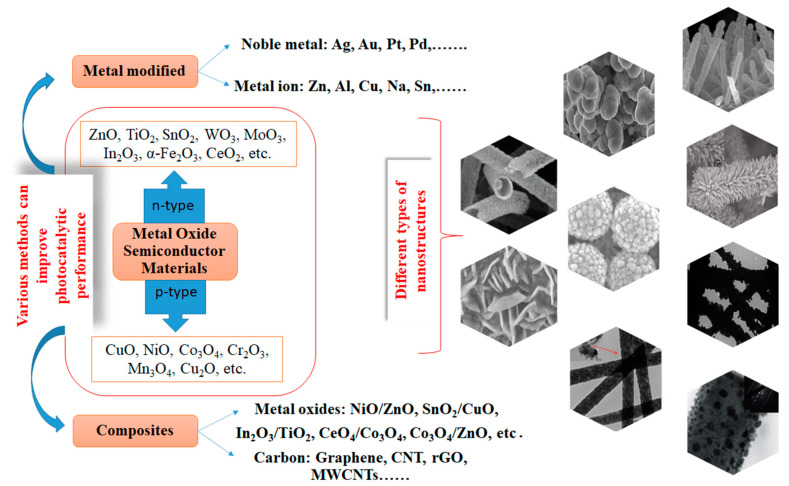
Different semiconducting metal oxide nanostructures used for photocatalytic applications.

**Figure 2:**
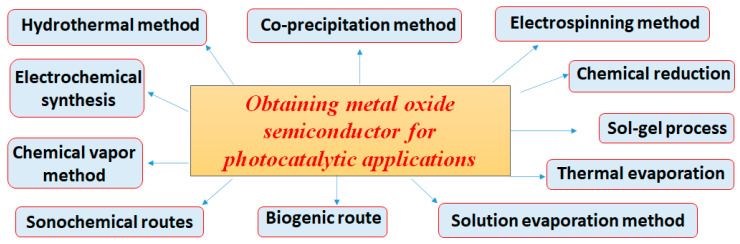
Methods of obtaining metal oxide nanostructured materials used as photocatalysts.

In recent years, a variety of nanostructures (Ns) with different shapes, such as spheres, cuboids, diamond-shaped nanoparticles, nanotubes, nanowires, nanofibers, etc. (Figure 1) were prepared using different methods (Figure 2) to obtain them and then tested for the degradation of different organic pollutants in wastewater [23,24].

It is known that the method and conditions of preparation of these materials can significantly improve photocatalytic performance by inducing some structural and morphological changes and obtaining materials with interesting shapes and sizes [25]. Moreover, the band-gap energies and charge carrier separation of metal oxide semiconductors are dependent on their size, crystal phase, and crystallinity. Li et al. [26] proved that the bandgap energy is inversely proportional to the size of the oxide semiconductor nanostructures, leading to good control of the synthesis conditions and implicitly of the photocatalytic performances. 

*(a) Precipitation technique* is one of the most common and versatile methods to prepare metal oxide semiconductor nanostructures. This method has a multitude of advantages, including simplicity of scale-up, low cost, control of synthesis conditions (moderate temperatures), and diversity of particle surface modifications [27]. On the other hand, the method presents some disadvantages due to the impurities that can precipitate with the product, and for hybrid oxide semiconductor materials, it is difficult to control particle size and morphology. For example, El-Kemary et al. [28] obtained ZnO semiconductor nanostructures of about 2.1 nm diameter by chemical precipitation method. The authors reported ciprofloxacin (C_0_ = 5 mg/L) degradation efficiency of 48% at pH = 10 in the presence of ZnO nanoparticles (20 mg/L). Recently, Dineshbabu et al. [29] evaluated the performance of photocatalytic degradation of tetracycline using binary (ZnO/g-C_3_N_4_, NiO/g-C_3_N_4_, ZnO/NiO) and ternary (ZnO/NiO/g-C_3_N_4_) photocatalysts prepared by the co-precipitation technique. It was found that the ternary system presents superior photocatalytic performances compared to the binary systems with an efficiency value of 91.49% and a degradation rate of 5.356 × 10^−2^ min^−1^. 

*(b) Sol–gel method* is another attractive method often used for the synthesis of oxide semiconductor nanostructures due to its low production cost, good repeatability, process simplicity, and low temperatures for synthesis, providing good optical properties to these nanoparticles by controlling the size and morphology of the material [30]. Over time, this method was improved by replacing solvents with water to reduce costs [31]. Malakootian et al. [32] prepared nanostructured photocatalysts based on TiO_2_ doped with Fe^3+^ by the sol–gel method for the degradation of the metronidazole drug pollutant. The authors studied the influence of different factors (pH, amount of photocatalyst, and degradation time) on metronidazole degradation under UVC irradiation. In addition, the pollutant removal efficiency reached values of 97% under optimal conditions (pH  =  11, catalyst dosage  =  0.5 g/L, and reaction time  =  120 min).

*(c) Hydrothermal synthesis* of metal oxide semiconductors is among the most used methods for developing nanomaterials with various morphologies. The method offers the possibility of tuning the morphological (size and shape) and structural properties by varying different synthesis parameters (reaction time, temperature, concentration of reaction compounds, pH, use of surfactants in the reaction medium, etc.). Several studies of photocatalytic properties using different forms of oxide semiconductor nanostructures obtained by this method were reported [33,34,35].

*(d) The electrospinning method* proved to be one of the most effective methods for obtaining one-dimensional oxide semiconductor materials. This is a simple, feasible, and low-cost technology method to fabricate one-dimensional (1D) nanostructures with a variety of diameters and a large surface area as compared to previously described methods. In general, the oxide semiconductor materials obtained by electrospinning are prepared in two stages as follows: (*i*) the first stage consists of the preparation of the intermediate products by the electrospinning technique (polymers + metal salts) and (*ii*) the second stage involves the calcination at different temperatures of the previously electrospun intermediates for the development the desired inorganic materials [36,37]. 

*(e) Biogenic synthesis* of the metal oxide nanostructures is a simple, cost-effective, non-toxic, and environmentally advantageous alternative to the chemical and physical methods. In the biogenic route, the toxic reactants are substituted by plant extracts, bacteria, fungi, or algae with lower risks of danger for the humans and environment [38,39,40]. Among the above-mentioned biological resources, plant extracts can be considered an ideal platform for the production of metal oxide nanoparticles because the bioactive compounds from extracts can act as reducing and stabilizing agents, monitoring the structure and characteristics of the material [39,41,42].

## 3. General Considerations on the Most Important Metal Oxide Semiconductors (ZnO, TiO_2_, CuO, etc.)

Zinc oxide (ZnO): Due to its exceptional chemical stability and remarkable photo-catalytic activity in the elimination of water pollutants, it is widely recognized that it serves as an outstanding photocatalyst. ZnO exhibits a substantial bandgap (3.37 eV) and, at room temperature, boasts a significant exciton binding energy (60 meV) [43,44]. Various nanostructures of ZnO, such as nanosheets, nanowires, nanobelts, nanorods, and complex hybrid structures, can be fabricated. Hollow spheres hold a special significance among these nanostructures due to their exceptional light-harvesting efficiencies, and significantly improved photocatalytic activity, along with their notable attributes of high surface area, low density, and excellent surface permeability. The physical and chemical characteristics of ZnO, including its enhanced electrochemical stability, remarkable oxidative capability, and low toxicity, render it a highly promising material for facilitating photocatalytic activity. While the photocatalytic approach offers numerous advantages, the high recombination rate of the photo-excited carriers in ZnO holds a drawback to both photocatalytic efficiency and the generation of photocurrent. Current research endeavors involve conducting thorough investigations to tune ZnO through the incorporation of noble, transition, and rare earth metals. The ultimate goal is to enhance the electrical and optical properties, thereby boosting the overall photocatalytic performance [45,46,47]. For example, Pascariu et al. [36,37] showed that ZnO nanostructures doped with different rare metals (La, Er, Sm) led to an increase in the photocatalytic performances for the degradation of some organic dyes. Furthermore, some studies have shown that hybrid semiconductor materials (e.g., CuS QDs@ZnO, ZnO/g-C_3_N_4_) and ZnO/carbon-based materials have superior photocatalytic properties [48,49,50]. Recently, Mukherjee et al. [50] reported a significant improvement in the photocatalytic performances for CIP degradation in the presence of carbon dots embedded on the surface of ZnO nanoparticles. They demonstrated that these materials possessed a photocatalytic degradation rate of 0.30 min^−1^ under optimum conditions (catalyst dosage of 0.6 g/L, natural sunlight, 12 mg/L of CIP initial concentration, 6.3 of pH, and 110 min of irradiation time), which was 3 times higher than for pure ZnO nanoparticles. In recent years, studies on the development of ZnO-based oxide semiconductor nanostructures obtained by green methods have been reported, as well as their use in the degradation of organic pollutants [51]. For example, Batterjee et al. [51] investigated the photocatalytic activity for the degradation of ciprofloxacin (C_0_ = 1.0 × 10^−4^ M) under UV light irradiation using ZnO nanoparticles obtained by green hydrothermal synthesis. Moreover, it was shown that the composite systems based on ZnO coupled with another semiconductor lead to the improvement of the photocatalytic performances for the degradation of drug pollutants [52]. In their study, Li et al. [52] found that the γ-Fe_2_O_3_@ZnO composite photocatalyst improved ciprofloxacin degradation by 92.5% as compared to the individually tested components γ-Fe_2_O_3_ (11.7%) and ZnO (52.4%).

*TiO_2_* is another oxide semiconductor used successfully in various applications such as sensors, dye-sensitized solar cells, electrochromic devices, lithium-ion batteries, cosmetics, pigments, catalysts, sunscreens, water splitting, etc. [53]. TiO_2_ can be obtained using a variety of techniques including the coprecipitation procedure followed by the calcination, hydrothermal, solvothermal route, sol−gel process, electrochemical procedure, electrospinning–calcination method, and so on [17]. Many studies demonstrated that the photocatalytic performances of TiO_2_-based materials are influenced by these synthesis methods, by modifying the structure, morphology, shape, and dimensions of these nanostructures [54,55,56]. Moreover, the significant interest in TiO_2_ is its affordability, making it a highly cost-effective option, in addition to its nontoxicity, exceptional stability, and widespread availability on a global scale [57]. Generally, TiO_2_ is found in an amorphous form or as three distinct crystalline polyforms: two tetragonal ones, rutile (P42/mmm) and anatase (I41/amd), and brookite (Pbca) as an orthorhombic phase [58]. Likewise, anatase and rutile phases are frequently used as catalysts for the degradation of various organic pollutants in wastewater [59]. Over time, commercial TiO_2_ which consists of a mixture of anatase (80%) and rutile (20%) phases having a surface area of 50 m^2^/g has been intensively studied [60]. Between these two phases, rutile is the most thermodynamically stable, whereas anatase is metastable and able to transform into rutile at high temperatures. In addition, the average particle size varies from the anatase phase (11 nm) to the rutile phase (≥50 nm) [54]. In general, TiO_2_ exhibits optical bandgaps of 3.0 and 3.2 eV for rutile and anatase phases, respectively. However, the reported literature values for brookite range between 3.13 and 3.40 eV in terms of bandgap values [61]. Titanium oxide being a wide bandgap material requires the use of UV light for irradiation in photocatalytic processes, but only 4–5% of sunlight covers the UV range. Nevertheless, extensive research has demonstrated that the anatase phase is extensively employed for the degradation of organic pollutants, primarily due to its superior photocatalytic activity and stronger adsorption affinity towards pollutant surfaces when compared to the rutile phase [54]. Most of the research on TiO_2_ photocatalysts proves that it solely absorbs UV light, thereby limiting their practical applications. To enable a more efficient harvest of visible light from solar irradiation, numerous techniques and strategies have been explored. These strategies include dye sensitizers, doping with metals or non-metals, heterojunction composites, deposition of noble metals, coupling with materials sensitive to visible light, morphology engineering, and incorporation of co-catalysts. These approaches aim to modify or transform metal oxides into visible-light-driven photocatalysts, expanding their range of applicability [11,62]. Recent studies in the literature have provided evidence that the synthesis of nanocomposites leads to the formation of diverse structures and shapes. This phenomenon contributes to an increased surface-area-to-volume ratio, as well as enhanced electrical, magnetic, and optical properties. As a result, these nanocomposites exhibit improved efficiency in the mineralization of organic pollutants. For example, Pascariu et al. [63,64,65,66,67] reported that by controlling the crystal structure (calcination temperature), the dopant percentage, the bandgap, the induction of microstructural defects, and the establishment of an optimal ratio between the anatase/rutile phases, nanostructures with improved photocatalytic performance can be developed. Additionally, the authors demonstrated that TiO_2_ can be doped with different metals to enhance its photocatalytic activity (Cu, Ag, La, Sm, Er, Sn, etc.). In related research [68], a correlation has been established between the morphology of certain graphene derivatives and titanium dioxide composites and their photocatalytic performances. These studies have highlighted a robust interaction between graphene and TiO_2_ particles, leading to a lower energy gap. In addition, TiO_2_ proved to be an efficient photocatalyst for the degradation of ciprofloxacin drug micropollutants under UV-visible light irradiation [63,64,67,69]. Recently, it was shown that by doping one-dimensional TiO_2_ semiconductor nanostructures with various transition metals and lanthanides, the photocatalytic performances for the degradation of ciprofloxacin under visible light exposure increase. According to Pascariu et al. [63,64], ciprofloxacin degradation in the presence of TiO_2_ nanostructures doped with lanthanides (Sm, Er, La) depends on their shape, size, and lanthanide nature. In addition, the authors found that 0.1% is the optimal amount for doping these materials to obtain a maximum degradation efficiency. Moreover, the intensification of the photocatalytic process in the presence of TiO_2_ photocatalyst doped with 0.1% Sm and calcined at 600 °C by adding H_2_O_2_ was evaluated [64]. The results showed that under optimal conditions, the rate constant was 4.292 × 10^−1^ min^−1^, and the half-life of the reaction decreased from 68 to 2 min, which is a remarkable result for the photodegradation process of down the visible light irradiation. Likewise, the TOC removal efficiency for CIP mineralization was 72.30%, with excellent reusability after five cycles. Duran-Alvarez et al. [70] reported a study on the degradation of ciprofloxacin under UV and simulated sunlight using TiO_2_ modified with mono- (Au, Ag, and Cu) and bi- (Au–Ag and Au–Cu) metallic nanoparticles. It was shown that the modified bimetallic photocatalysts presented the highest percentage of mineralization, and the degradation rates were up to 2.3 × 10^−2^ min^−1^. Moreover, the photodegradation of ciprofloxacin using nanocomposite materials based on Z-scheme TiO_2_/SnO_2_ as photocatalysts was recently reported by Costa et al. [69]. The authors reported an efficiency of up to 92.8% with a degradation rate of 2.24 × 10^−2^ min^−1^ and a half-life of 30.9 min after under UV light irradiation. In another study, Malakootian et al. [71] investigated ciprofloxacin degradation using TiO_2_ nanoparticles immobilized on a glass plate under UV light. The study aimed to solve the difficulties related to the separation of catalysts from the solution, as well as the reuse of nanoparticles.

*CuO* is a semiconductor oxide that has gained special attention in recent years due to its potential applications, such as gas sensors, dye-sensitized solar cells, paints, plastics, filters and textiles, antimicrobial properties, catalysis, etc. [72,73]. Among all metal oxides, cupric oxide (CuO) has been extensively studied due to its outstanding properties as a p-type semiconductor with a narrow bandgap (1.2–2.0 eV) and its numerous interesting properties such as thermal superconductivity, photovoltaic properties, high stability, and antimicrobial properties [74]. Furthermore, the variation of the bandgap can be attributed to the quantum effect due to the size observed in different CuO nanostructures. Various techniques can be employed to characterize the optical properties of nanomaterials or estimate the specific bandgap. The reaction rates can be influenced by particle size and shape, as they impact the amplitude of the bandgap and the diffusion length of photo-generated charge carriers from the bulk to the surface-active sites. The value of the bandgap energy, in turn, plays a crucial role in determining the photonic efficiency and the wavelength interval of the electromagnetic spectrum that can be harnessed during degradation reactions. CuO in its pure form exhibits a bulk bandgap of 1.2 eV, but through appropriate synthesis methods, this value can be increased up to 3.57 eV in the nanoscale size domain. Various synthesis methods (co-precipitation, hydrothermal, sol–gel process, chemical vapor deposition, sonochemical routes, electrochemical method, electrospinning technique, etc.) allowed scientists to develop materials with different shapes (nanowires, nanocubes, nanoribbons, nanoflowers, nano-octahedra, nano-shuriken, nanofiber, etc.), morphologies, and sizes [75,76]. The copper salts (chloride, nitrate, sulfate, or acetate) could be used as precursors to obtain the CuO nanostructures. CuO having a monoclinic crystal structure possesses special physical and chemical properties (large surface area, adequate redox potential, good electrochemical activity, thermal superconductivity, and excellent stability in solutions). The photocatalytic activity of nanoparticles is significantly influenced by their crystalline nature. Nanoparticles with high crystallinity and favorable charge transport characteristics exhibit superior catalytic performance. Leveraging their unique physical and chemical properties, CuO nanoparticles have proven to be effective in removing certain environmental contaminants such as dyes and antibiotics from wastewater. As a semiconductor catalyst, cupric oxide nanoparticles can be activated through visible light irradiation to degrade various pollutants. However, there are limited reports on the photocatalytic degradation of ciprofloxacin (a widely used fluoroquinolone antibiotic) under visible light irradiation. Along this, Cu/CuO composites are of great interest due to their non-toxic and environmentally friendly wide range of applications in advanced oxidation processes (AOPs). These composites enable the degradation of various pollutants in contaminated waters, particularly through photocatalytic pathways [77,78,79]. Research studies have shown that doping CuO with different metals or combining it with materials such as TiO_2_ can enhance photocatalytic activity by effectively reducing the recombination effect of photogenerated electron/hole pairs [80,81]. Moreover, the photocatalytic properties of ciprofloxacin degradation were improved by Zn doping of Cu_2_O nanoparticles prepared by the solvothermal method [82]. Yu et al. showed that the surface morphology of Zn-doped Cu_2_O particles changed significantly as the amount of ZnCl_2_ dopant was modified. Additionally, the size of Cu_2_O nanoparticles doped with Zn decreased, the specific surface area increased, and the photodegradation efficiency improved.

## 4. Alternatives for Improving the Photocatalytic Performance of Metal Oxide Semiconductor

Metal oxide semiconductor materials used in photocatalytic degradation processes can be classified into n-type materials that include ZnO, SnO_2_, TiO_2_, WO_3_, MoO_3_, In_2_O_3_, α-Fe_2_O_3_, CeO_2_, etc., and p-type materials with the following structures: CuO, NiO, Co_3_O_4_, Cr_2_O_3_, Mn_3_O_4_, Cu_2_O, etc. [83,84,85,86]. Unfortunately, the practical use of metal oxide nanomaterials as photocatalysts encounter some difficulties due to rapid electron–hole recombination rate and insufficient light absorption range which can lead to restrictions in the generation of powerful oxidizing species, namely, hydroxyl (•OH) and superoxide (O_2_•^−^) radicals, singlet oxygen under light irradiation. These oxidative species present a high potential for degradation of the contaminants from aquatic environments. To overcome this problem, researchers have proposed different methods to improve the photocatalytic performance, such as modifying these semiconductors by including noble metals, doping with different transition and rare metals, developing composite nanostructures consisting of p-n types of semiconductors, as well as producing nanomaterials based on semiconductors/carbon. In this way, the light absorption ability and the separation efficiency of charge carriers are improved.

Numerous studies [86,87,88,89] have been reported on the surface modification of semiconducting metal oxide nanostructures using noble metals in order to improve photocatalytic performance. The most common noble metals used to modify the metal oxide semiconductor as a photocatalyst are Au, Ag, Pt, and Pd, respectively [86,87,88,89]. Furthermore, semiconductor materials doped with rare metals improved the photocatalytic performance for the degradation of different organic pollutants in wastewater. For example, Manasa et al. [90] reported the photocatalytic degradation of ciprofloxacin (C_0_ = 10 mg/L) under sunlight irradiation using Ce-doped TiO_2_ as a photocatalyst. Likewise, Pascariu et al. [36,37,63,64] demonstrated that doping ZnO and TiO_2_ nanostructures with La, Er, and Sm can enhance the photocatalytic activity of some organic pollutants (dyes and drugs). In addition, transition metals such as Ni, Cu, Fe, V, Co, etc., have been used to modify oxide semiconductors in order to improve the photocatalytic activity and extend their performance in the visible region of the electromagnetic spectrum [44,91,92]. These dopants in semiconductor materials can improve the density of active centers and surface defects on semiconductor nanostructures, leading to enhanced photocatalytic activity.

Another method of improving catalytic performance can be achieved by coupling two semiconductors or by combining a semiconductor with carbon nanomaterials [93,94]. As an example, p-n heterojunction may occur at the interface between the p- and n-type semiconductors, where the electrons located in the conduction band corresponding to the n-type semiconductor will jump on the valence band of p-type semiconductor through the heterojunction due to its lower energy. In this way, a depletion region will occur at the p–n heterojunction interface due to electron–hole recombination. Alshaikh et al. [95] obtained Co_3_O_4_/ZnO p–n heterojunctions with good photocatalytic properties for ciprofloxacin degradation under visible light irradiation. Likewise, Shen et al. [96] prepared a composite heterostructure using CeO_2_ in different ratios (3, 5, and 10%) and Co_3_O_4_ for the degradation of ciprofloxacin. The best photocatalytic performances (87.8%) for CIP degradation (C_0_ = 5 mg/L) were obtained for 5% CeO_2_/Co_3_O_4_, after 50 min of visible light using 0.5 g/L catalyst. The increase in photocatalytic activity for ciprofloxacin degradation (15 mg/L) due to the synergism between CeO_2_ and ZnO was also observed by Wolski et al. [97]. Recently, Zhou et al. [98] reported an enhanced photocatalytic degradation of ciprofloxacin (C_0_ = 12 mg/L) over Bi_2_MoO_6_/g-C_3_N_4_/BiFeO_3_ heterojunction photocatalyst under visible light exposure. 

These new oxide semiconductor materials form on the surface and at the interface oxygen vacancies, microstructural defects, heterojunctions, and mesopores, which ensure the formation of more active sites for the enhancement of pollutant degradation reactions. Additionally, heterojunctions are formed at the interface between the two semiconductor materials and can accelerate the electronic transfer between the particles leading to an increase in the reaction rate of the photocatalyst. 

The latest series of composite materials and the novelty of today is the coupling of *semiconductor nanostructures with carbon nanomaterials* [99,100]. The most promising carbon-based materials are graphene and carbon nanotubes. These photocatalysts based on carbon nanomaterials showed improvements in photocatalytic performance due to many active sites (such as oxygen functional groups, vacancies, and defects) formed at the interfaces. Remarkable progress has been reported for a lot of composite nanostructures based on semiconductors and carbon nanomaterials in different structures (nanosheets, nanoparticles, spheres, thin sheets, nanorods, nanocomposites, s.a.), among which include ZnO–NiO/rGO [101], Fe_3_O_4_–NiO/rGO [102], TiO_2_/rGO [103], ZnO/GO [104], and so on.

## 5. Photocatalytic Degradation of Ciprofloxacin Pollutant from Water

**Figure 3:**
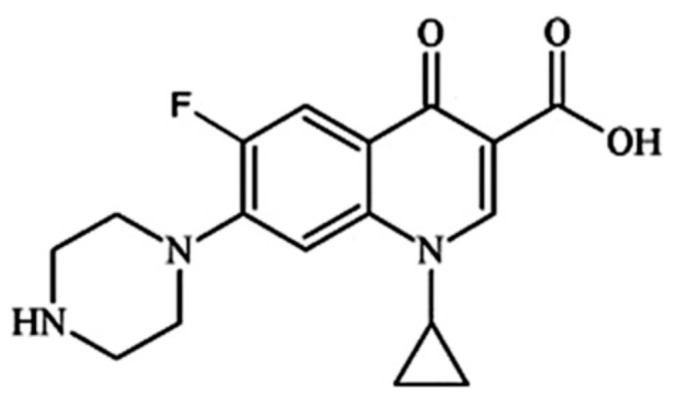
Chemical structure of ciprofloxacin (C_17_H_18_FN_3_O_3_).

The presence of antibiotics in aqueous environments can generate major risks due to the increased bacteria resistance to conventional wastewater treatments. Antibiotics are chemotherapeutic agents that cure bacterial infections [105]. Different techniques such as flocculation, physical adsorption, or chemical oxidation were applied to remove the antibiotic contaminants from the aquatic ecosystems. However, these procedures usually present some limitations including the incomplete elimination of the antibiotics and the formation of some post-treating sediments resulting from the used chemical agents and polymer electrolytes. Furthermore, the photocatalytic methods for the removal of antibiotics became more attractive due to their relative eco-friendly character, presenting high efficient reaction conditions, nontoxicity, and cost-effectiveness during the degradation of antibiotics. Thus, the antibiotics can be effectively decomposed leading to greater interest in finding adequate photocatalysts for antibiotic degradation. Ciprofloxacin (1-cyclopropyl-6-fluoro-4-oxo-7(piperazin-1-yl)-quinoline-3-carboxilic acid) (Figure 3) is a second-generation fluoroquinolone antibiotic, which consists of a quinolone structure and a piperazine unit [106]. Ciprofloxacin (CIP) is one of the clinical antibiotic compounds frequently detected in the pharmaceutical effluents present in contaminated waters, with different ionizable groups leading to a complex acid-base equilibrium, which makes its degradation behavior hard to predict [107]. 

Moreover, ciprofloxacin is a well-known drug indicated for human and animal infections, occupying the fourth position in the European antibiotic market [108]. A recent study performed in Europe (2017) has shown that from the whole consumption of quinolones, expressed in DDD per 1000 inhabitants per day, a percentage of 90% can be attributed to the four following substances: ciprofloxacin (48.6%), levofloxacin (28.8%), norfloxacin (10.4%), and moxifloxacin (7.2%) The consumption of quinolones in the European community varied between the countries with the highest rate (2.86 DDD per 1000 inhabitants per day, Bulgaria) and the lowest (0.35 DDD per 1000 inhabitants per day, Norway) [108]. Ciprofloxacin is among the most utilized chemotherapeutic agents which can alter the DNA gyrase and topoisomerase IV of different varieties of Gram-positive and Gram-negative bacteria, thus, preventing cellular replication [109]. Ciprofloxacin is extensively used to treat various infectious diseases namely gastrointestinal infections, complicated urinary tract infections, sexually transmitted infections, respiratory tract diseases, and skin infections [110]. The increased production of ciprofloxacin and the appearance of new contagious diseases have determined a higher accumulation of CIP in the ground waters and soil [111]. Ciprofloxacin is the most commonly detected fluoroquinolone antibiotic in contaminated waters by pharmaceutical effluents, up to 31 mg/L [112]. The presence of ciprofloxacin residues in aquatic ecosystems beyond a certain limit can determine physico-chemical modifications in the high surface areas of soil components (clay minerals and metal oxides) and can affect the ability to monitor the mobility and bioavailability of other contaminants and micronutrients [113]. However, for micro-organisms, the release of ciprofloxacin into the environment at high levels can induce some chromosomal mutations of the native bacteria leading to ciprofloxacin-resistant bacterial strains [114]. In this way, in order to diminish the number of bacterial infections, a higher content of antibiotics will be necessary to the detriment of public health [115]. In addition, the utilization of ciprofloxacin, even at low concentrations, can induce transient changes in microbial activity, which might be essential for their productivity [116]. Other aspects such as toxicological effects on non-intended pathogens, alteration of structure, and dissemination of algal communities have also been related [116]. Mechanism and performance evaluation of photocatalysts for ciprofloxacin removal by the catalytic processes is initiated by the irradiation of the photocatalyst with photons having energy corresponding to or greater than its bandgap. This process determines the generation and subsequent transfer of electron–hole pairs to the photocatalyst surface [117]. The mechanism of photocatalytic degradation of antibiotics is dependent on the formation of free radicals and active oxygen species. Physico-chemical characteristics such as morphology and surface areas are dominant factors in the performance of catalysts during the photodegradation process. Moreover, morphological properties and surface area play an important role in the catalyst performances during photodegradation investigations. Photocatalysis has many advantages as compared to other conventional methods, namely, efficient degradation of contaminants, wastewater remediation, environmental protection, clear surface of different materials, air purification, and no secondary pollution. According to Table 1 [21,61,64,90,95,96,97,118,119,120,121,122,123], various studies have investigated ciprofloxacin degradation in the presence of nanostructured oxide materials.

**Figure 4:**
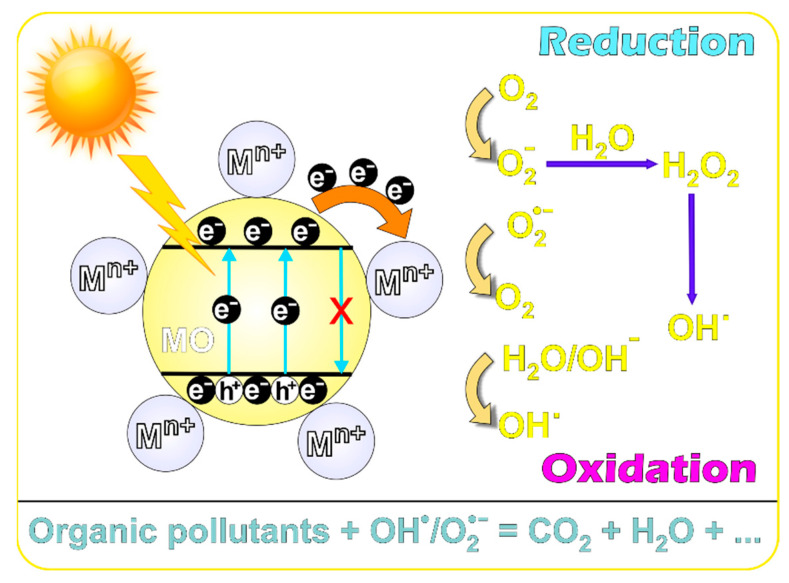
Possible mechanism of organic pollutants degradation in the presence of metal ions-doped metal oxide photocatalysts [114].

Additionally, each method of obtaining these materials can improve their photocatalytic activity. Furthermore, a degradation mechanism of some organic contaminants (CIP) in the presence of metal oxide nanostructures doped with metal ions (e.g., M^2+^) can be shown (Figure 4). Electrons are excited from the valence band (VB) to the conduction band (CB) when UV or visible light of a certain wavelength is absorbed by the photocatalyst. This generates positive charge carriers (holes, h^+^) in the VB of the metal oxide catalysts. These positive charges (h^+^) in VB produce free hydroxyl radicals (•OH) from H_2_O molecules. In addition to this, the electrons generated (e^−^) in CB are captured by oxygen molecules and super oxygen radicals ($O_{2}^{•-}$) are produced. Additionally, the photogenerated holes can react with the water molecules producing •OH radicals. Due to their high oxidation ability, the •OH radicals contributed to the decomposition of the organic contaminants adsorbed on the catalyst surface. Finally, the organic pollutants will be removed by these strong reactive species resulting in harmless CO_2_, H_2_O, and other by-products depending on the pollutant type [119,124]. 

## 6. Factors Affecting the Photocatalytic Degradation of Oxide Semiconductor Materials

The photodegradation of organic pollutants in the presence of metal oxide catalysts is influenced by several factors. These factors encompass the initial concentration of the pollutant, the composition, and quantity of the photocatalyst, as well as the morphology, shape, and surface properties of the photocatalyst. Additionally, the temperature of the solution, pH levels, light intensity, and duration of irradiation also play significant roles in this process. Among these factors, some operational parameters significantly affect photocatalytic degradation and efficiency, as discussed below.

*The structure and morphology of metal oxide nanostructures* play a major role in the characteristics and photocatalytic performances. Shape/size (0D, 1D, 2D, 3D), surface properties (porosity, roughness, etc.), aspect ratio, exposed crystal facets, and particle size are attributes of morphology. It should be mentioned that the photocatalytic degradation of organic pollutants can be divided into five main steps: (1) transfer of pollutant in the fluid phase to the surface; (2) pollutant diffusion; (3) adsorption of pollutant molecules on the catalyst surface; (4) desorption of the products; and (5) removal of products from the catalyst surface [125]. Therefore, the surface area is one of the extremely important parameters for tuning the photocatalytic activity. Moreover, a larger surface area of metal oxide nanostructures provides a large number of photocatalytic active sites and improved pollutant degradation efficiency. Likewise, the surface’s properties are highly dependent on the shape, size, and nature of the surface (porosity, toughness, etc.). Many studies of photocatalytic properties have been reported using different shapes and sizes of semiconducting metal oxide nanostructures as catalysts [56,62,67,126]. For instance, in a study conducted by Fan et al. [56], it was shown that the degradation efficiency of Rhodamine B dye under UV light irradiation in the presence of hierarchical TiO_2_ nanostructures was influenced by their different shape. Another group [126] showed the importance of the effect of size and shape (cubic, spherical, ellipsoidal nanoparticles, and nanoparticles) on the degradation of methyl orange dye. However, it is known that the anatase form is superior to the other two phases in terms of photocatalytic activity. For this reason, there are studies to optimize these phases to improve the photocatalytic activity of different organic pollutants. Recently, Pascariu et al. [65,67] demonstrated that to obtain photocatalysts with outstanding performance, it is essential to control the crystal structure by calcination temperature. According to one of the works [67], the study evaluated the impact of Sn content and calcination temperatures on the structures, functionalities, and degradation efficiency of the drug pollutant ciprofloxacin under visible light. They found that the most efficient catalyst is the 1.5% Sn-doped TiO_2_ material calcined at 500 °C (k = 9.685 × 10^−2^ min^−1^) followed by the same sample but calcined at 600 °C (k = 6.392 × 10^−2^ min^−1^). Furthermore, it was observed that the rate constant for the photodegradation of ciprofloxacin (CIP) increased with each successive addition of Sn, and the calcination process was conducted at a temperature of 500 °C.

*Photocatalyst dosage*: the catalyst loading range for photodegradation processes varies with the specific system and is crucial to the degradation process. However, a commonly observed range for catalyst loading is between 0.04 and 5.0 g/L. The relationship between catalyst loading and photodegradation efficiency is widely known, as higher catalyst loading leads to an increase in the active sites that come into contact with the pollutant, consequently increasing the photodegradation efficiency. However, there is a limit beyond which the maximum efficiency of the catalyst decreases. This decrease can be attributed to various factors, such as increased turbidity of the solution, which in turn reduces light transmission. The formation of hydroxyl and superoxide radicals leads also to an increase in the exposed active reaction sites. The catalyst in excess determines the decrease in the photodegradation efficiency due to the increase in the solution turbidity and the decrease in the light penetration as well as a lower dispersion of the nanomaterial in the solution. Additionally, the agglomeration of the nanoparticles can occur and the catalyst’s ability to generate reactive oxygen species is lower, increasing the surface inactivation due to the particle collisions [50,119]. For example, Georgaki et al. [127] studied the effect of catalyst loading (between 0.05 and 0.5 g/L) for ZnO and TiO_2_ materials at an initial pollutant concentration of 10 mg/L. Recently, Pascariu et al. [63] reported the effect of the catalyst dosage of 0.1% La-doped TiO_2_ 1D nanostructures varying the catalyst between 0.2 to 1 g/L for MB dye degradation under visible light irradiation. Kinetic studies of the photocatalytic process showed rate constant values varying between 7.625 × 10^−3^ and 2.179 × 10^−2^ min^−1^, depending on the amount of catalyst. In addition, the best catalyst sample (0.1% La-doped TiO_2_) was tested for the photodegradation of the ciprofloxacin drug micropollutant, under visible light irradiations. The authors showed that for the photodegradation of CIP (C_0_ = 10 mg/L), the rate constant was 1.981 × 10^−1^ min^−1^ and a degradation efficiency of about 100% for the following experimental conditions: 0.6 g/L catalyst dosage, reaction temperature of 23 °C, and pH of 6.0. In another study [128], the authors demonstrated the importance of catalyst dosage for chloramphenicol (CAP) drug pollutant degradation. The influence of photocatalyst loading on CAP degradation efficiency was investigated in the range from 0.1 to 1.0 g/L. They find that at 0.5 g/L of catalyst, the equilibrium is reached, and above this value, the efficiency decreases. According to Eskandari et al. [129], changing the quantity of ZnO photocatalyst from 0.05 to 1.5 g/L can increase the degradation efficiency of ciprofloxacin from 50% to 90%.

*Initial pollutant concentration:* Numerous studies have been carried out to evaluate the influence of the initial concentration of the pollutant on its degradation process [123,130,131]. For instance, Isai et al. [130] reported that the degradation rate of MB dye is contingent upon two factors: the potential formation of hydroxyl radicals (•OH) on the catalyst surface and the reactivity between MB molecules and •OH radicals. The authors found that the photocatalytic activity of the Fe-doped ZnO catalyst decreases as the dye concentration increases. Due to the presence of more dye molecules attached to the catalyst surface, the reaction between MB-OH^•^ is slowed down, limiting the active sites of the catalysts and decreasing the production of •OH. In addition, it is known that for the degradation of a dye molecule, a certain number of hydroxyl radicals, a certain light intensity, a certain dosage of the catalyst, and an irradiation time are necessary. Therefore, increasing the initial concentration of pollutants leads to a decrease in photodegradation efficiency [131]. In a recent study conducted by Pascariu et al. [123], the photodegradation process of ciprofloxacin was optimized by considering both the pollutant concentration and the dosage of the catalyst in the presence of 0.1% Nd-doped ZnO photocatalyst. The researchers found that the removal efficiency was about 100% (after 120 min) with the rate constant of k = 5.291.10^−2^ min^−1^ and a reaction half-life (τ = ln2/k) of 13 min for the optimal conditions.

*Light wavelength and intensity:* The photocatalytic degradation process depends on the wavelength and intensity of the light with which the materials are irradiated. In addition, it is crucial to consider the energy bandgap of each semiconductor material. Many researchers [132,133] found that the rate constant of the photocatalytic reaction is determined by the intensity of the light. The most utilized light sources in the photodegradation of ciprofloxacin are UV lamps in the ranges UVA (315–380 nm), UVB (290–315 nm), and UVC (200–290 nm). The photodegradation efficiency for ciprofloxacin can be expressed in the following order: UVA < UVB < UVC [134], taking into account that the UVC sources emit photons with high energy. In this case, a great number of e^−^/h^+^ pairs will be generated and more reactive radical species appear in the system to degrade the target contaminant.

*Temperature* is another important parameter in the evaluation of photocatalytic activity. The majority of photocatalytic reactions take place at room temperature. However, studies have suggested that photocatalytic activity can be determined at temperatures between 20 and 80 °C. For instance, in a study by Tekin et al. [135], the influence of temperature on the rate constant for the degradation of Orange G dye was evaluated using ZnO and Ag/ZnO thin film photocatalysts. The researchers observed an increase in the photodecomposition rate as the reaction temperature increased within the range of 20–50 °C. Another study [136] showed that the reaction rate is dependent on temperature, and increases from 0.0111 min^−1^ (30 °C) to 0.0161 min^−1^ (50 °C) for the degradation of methyl orange using 1 g/L of 10% Co–ZnO photocatalyst. Mozia et al. [137] reported that at very low temperatures (below 0 °C) the photocatalytic activity decreases, leading to a limitation of the reaction rate which becomes desorption of the final product from the catalyst surface. The reaction rate of photocatalysis is reduced at high temperatures (above 80 °C), due to exothermic contaminant adsorption. The photocatalytic degradation of the ciprofloxacin presents a decrease at higher temperatures due to the increased thermal stability of the fluoroquinolone moiety [122]. In addition, Chen et al. [138] investigated the effects of reaction temperature on the photocatalytic activity of Pd/TiO_2_ and Cu/TiO_2_ catalysts. The study demonstrated that photocatalytic activity is affected by the reaction temperature and the type of materials.

*pH* is a key parameter in the processes of photocatalytic degradation of pollutants, being very important in the development of active oxygen species. Thus, pH will affect the surface energy, size, catalyst charge, aggregation, valence, and conduction band positions of the photocatalyst. The solution pH can significantly influence the photocatalytic efficiency of CIP, since the zwitterionic structures are dominant at pH between 5.5 and 7.5, whereas the cationic species are present at pH values < 5.5 owing to protonation at –NH group and the anionic species are formed at pH > 7.7 [50,139]. The pH effect on the photocatalytic process is determined by many variables, namely, the electrostatic interactions between the metal oxide surface, solvent medium, and radicals appearing during the catalytic reaction and we shall have different kinetics and degradation mechanisms and reaction products. In this way, the structural modifications determined by changing pH could improve or delay the CIP photolysis in the presence of metal oxide nanoparticles. It was observed for the TiO_2_/montmorillonite system that at pH 5, the photocatalytic degradation of CIP was significantly improved [134], while for zinc oxide nanoparticles the photodegradation rate was increased at higher values of pH [28]. Using Fe-doped ZnO nanocomposites as the photocatalyst, a photodegradation efficiency of around 65% was obtained at pH = 9, while at pH = 2 the lowest efficiency was found [122]. However, the maximum photocatalytic degradation was observed at pH 4 for CIP catalyzed by Mn/Co composites due to a low electrostatic interaction between the negative surface-charged catalyst and ciprofloxacin [140]. Additionally, the photodegradation yield of CIP at pH 9 was low using carbon dots included in ZnO nanostructures as a catalyst, and the best yield was found to be at pH 6.3, which is the natural pH of a ciprofloxacin solution [50]. The higher photodegradation of CIP at basic values of pH can be due to the formation of hydroxyl ions (·OH), which present a high oxidation ability leading to ciprofloxacin oxidation [122]. Similar effects of pH were reported by Chanu et al. [131] on the photocatalytic activity (between 5 and 12) for MB degradation in the presence of Mn-doped ZnO photocatalysts. The degradation efficiency exhibited an increase as the pH was increased from 5 to 12. The highest value (99%) was achieved at pH = 10 after 120 min of UV light irradiation. Moreover, Raja et al. [141] evaluated the effect of pH (in the range from 3 to 8) on Eosin Yellow photodegradation using 0.375 g/L Zn_2_SnO_4_-V_2_O_5_ as a catalyst under 180 min of irradiation time. They showed that the degradation efficiency increases between pH 3 and 4, and a further increase from 5 to 8 leads then to a decrease in the degradation efficiency.

*Hydrogen peroxide (H_2_O_2_) added into the system:* By adding hydrogen peroxide (H_2_O_2_) to the system, it leads to an intensification of the photodegradation process of organic pollutants. For instance, Pascariu et al. [64] studied the influence of the catalyst dosage and the initial concentration of hydrogen peroxide for methylene blue and ciprofloxacin degradation using 0.1% Sm-doped TiO_2_ nanofibers. The authors showed that the rate constants increased from 10^−2^ min^−1^ to 10^−1^ min^−1^, and the half-life of the reaction decreased dramatically from 68 min to 2 min, indicating a remarkable photocatalytic process under visible light irradiation. Moreover, these results were obtained under the optimal conditions found by them, namely, an amount of catalyst was 0.9 g/L and 0.11 M of H_2_O_2_ added to the system. The workable activity of the photocatalysts for ciprofloxacin degradation is determined by the stability of the photocatalytic sample during successive cycles of degradation. The degradation pattern in five photocatalytic cycles practically does not change, since the photocatalytic capacity was found to be about 99% and the XRD data are similar to the initial photocatalyst. Likewise, Kousar et al. [142] reported an improvement in photocatalytic performance after adding 0.8 mL of H_2_O_2_ for Reactive Yellow 160A dye under UV light irradiation using TiO_2_ as a photocatalyst, achieving a color removal efficiency of 90.40% compared to 28% without H_2_O_2_. 

## 7. Conclusions and Perspectives

The presence of antibiotics in the environment can contribute to a number of problems by fostering the widespread development of antimicrobial resistance. In this regard, metal oxide nanostructures (MONs) are of increasing interest in the field of environmental protection. Considering the large number of works based on metal oxide semiconductors, the present study aims to review and summarize the progress in this field for the degradation of organic pollutants, especially drugs. The important applications of the metal oxide nanoparticles for the remediation of pharmaceutical wastes under visible light irradiation were pointed out. The preparation of the new photocatalytic materials based on metal oxide semiconductors for the degradation of the organic pollutants containing antibiotics with an excellent response to UV and visible irradiation should be further than the first importance. Further studies are still necessary which are included highlights on the degradation mechanisms, evaluation of the toxicity of secondary products, and the development of new low-cost materials with controlled morphology, porosity, and optical properties. All these are important to maximizing the photocatalytic performance of the new materials. Moreover, the incorporation of these nanostructures into polymer matrices and testing on pharmaceutical wastewater sources are still needed.

## Figures and Tables

**Table 1:** Ciprofloxacin degradation in the presence of various nanostructured oxide materials.

Catalyst	Synthesis Method	Pollutant, C_0_	Catalyst Amount, pH	Time, Light Source	η (%), k (min^−1^)	Ref.
ZnO/FeTiO_3_	Sol–gel	CIP, 10 mg/L	1 g/L, 7	180 min, UV–Vis	- 0.0390	[118]
ZnO/Co_3_O_4_	Sol–gel	CIP, 10 mg/L	2.4 g/L	30 min, Visible (300 W Xe lamp)	100, 0.2	[95]
TiO_2_/Ce	Sol–gel	CIP, 40 mg/L	0.5 g/L, 5.5–6	180 min, UV (257 nm)	90–93, -	[90]
TiO_2_/WO_3_	Sonochemical–microwave	CIP, 20 mg/L	0.5 g/L	120 min, UV 120 min, sunlight	100, 0.133	[119]
					96, 0.034	
TiO_2_	Solvothermal	CIP, 10 mg/L	0.1g/L	120 min, UV (16 W)	57	[120]
CuO	Precipitation method	CIP, 10 mg/L	5 g/L	300 min, visible (400 W)	60	[121]
ZnO/CeO_2_	Precipitation method	CIP, 15 mg/L	0.25 g/L, 3.2	60 min, UV	0.0130	[97]
ZnO/Fe	Precipitation method	CIP, 10 mg/L	0.15 g/L, 9	210 min, visible	65, -	[122]
CeO_2_/Co_3_O_4_	Precipitation method	CIP, 5 mg/L	0.5 g/L	50 min, Visible (300 W Xe lamp)	87.8	[96]
TiO_2_/N	Precipitation method	CIP, 30 mg/L	1 g/L, 5	120 min, UV (257 nm)	94.5	[21]
TiO_2_/La (0.1%)	Electrospinning	CIP, 10 mg/L	0.6 g/L	300 min, visible (400 W)	99.5	[63]
TiO_2_/Sm (0.1%)	Electrospinning	CIP, 10 mg/L	0.9 g/L	300 min, visible (400 W)	~99	[64]
TiO_2_/Er (0.1%)	Electrospinning	CIP, 10 mg/L	0.9 g/L	300 min, visible (400 W)	~99	
ZnO/Nd (0.1%)	Electrospinning	CIP, 6 mg/L	0.9 g/L, 6	120 min, visible (400 W)	~99, 0.053	[123]
